# BCG instillations can mimic prostate cancer on multiparametric MRI

**DOI:** 10.1590/S1677-5538.IBJU.2017.0621

**Published:** 2018

**Authors:** Pablo Garrido-Abad, Miguel Ángel Rodríguez-Cabello, Cristina González-Gordaliza, Roberto Vera-Berón, Arturo Platas-Sancho

**Affiliations:** 1 Department of Urology, Hospital Universitario Sanitas La Moraleja, Madrid, Spain; 2 Department of Radiology, Hospital Universitario Sanitas La Moraleja, Madrid, Spain; 3 LABCO (SYNLAB) Pathology, Madrid, Spain

## CASE DESCRIPTION

**Figure 1:**
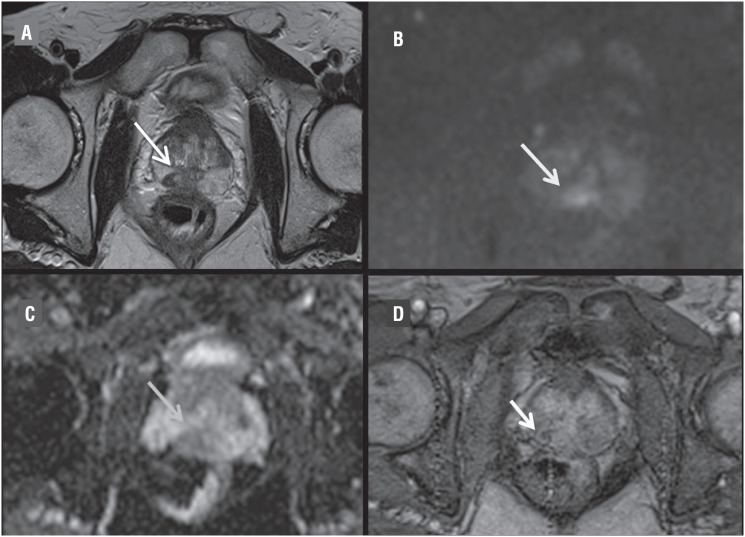
A) Axial T2-weighted image with a round hypointense focal lesion (6mm) in the right posteromedial peripheral zone at the midgland level (arrow). B) On diffusion-weighted imaging with high b value (1000), a focal markedly hyperintense lesion (arrow), with markedly hypointense (arrow) value on ADC map (0.8×10-3mm2/s), consistent with a PI-RADS 4 lesion. c) is described. On Dynamic contrast Enhanced image an enhancement (arrow) of nodular lesion is showed (D).

**Figure 2:**
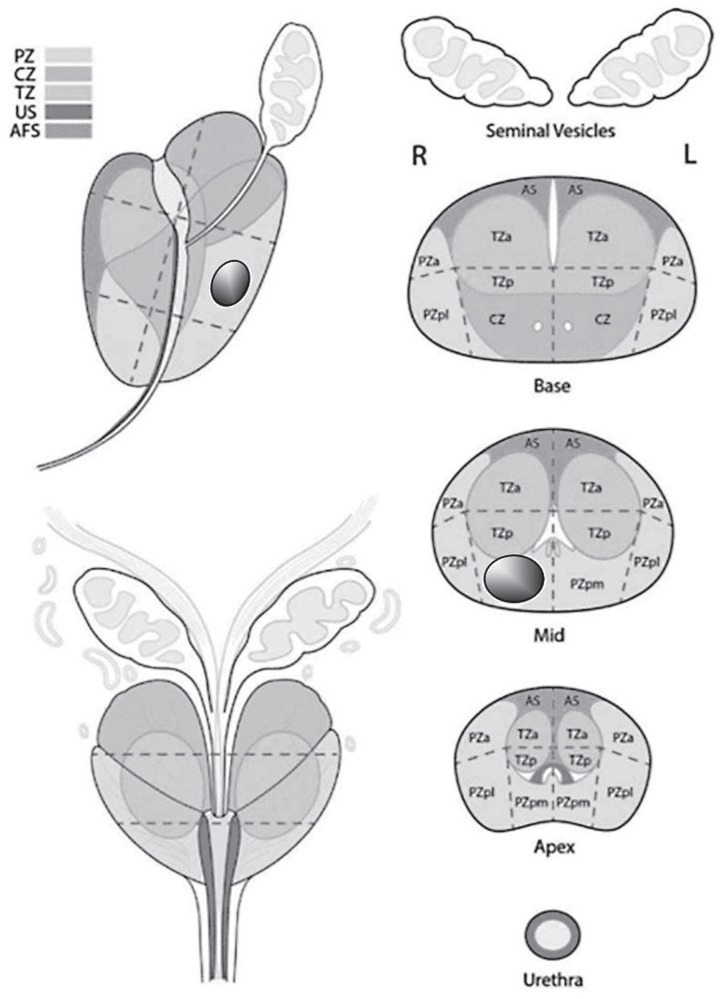
PI-RADS anatomic division document with marked suspicious PI-RADS 4 lesion in the right posteromedial peripheral zone at the midgland level.

**Figure 3:**
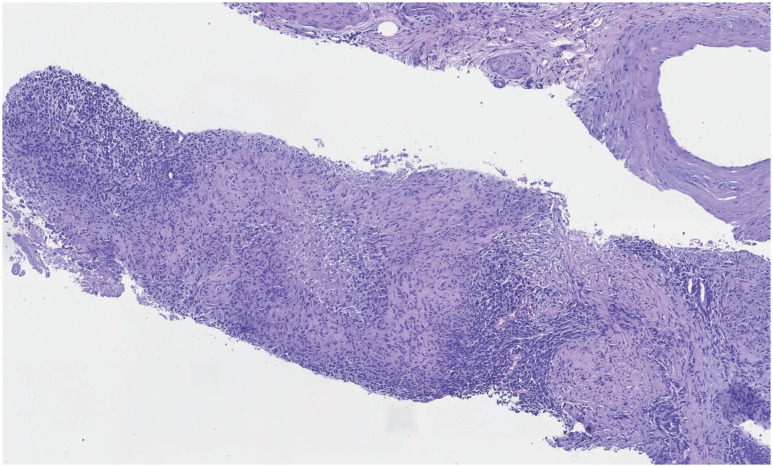
H&E-stained slide (10x) from a prostate needle core biopsy with granulomatous prostatitis showing epithelioid cells, multinucleated giant cells and lymphocyte infiltration.

A 63-year-old man presented with rising PSA that was 6.13ng/mL on last visit. He had a negative prostate biopsy 1 year ago, and is currently being treated with intravesical Bacillus Calmette-Guérin (BCG) instillations for pT1G3 bladder carcinoma. Multiparametric magnetic resonance (mpMRI) was carried out using a 1.5T system (Signa Excite, GE Healthcare) with a PI-RADS v2 score of 4 for diffusion-weighted imaging (DWI) in the right posteromedial peripheral zone at the midgland level (Figure-1). Thus, a systematic 42-core, sector-guided transperineal prostate biopsy, with additional cognitive targeted biopsy of the suspicious lesion was performed (Figure-2). Histological findings showed typical features of granulomatous prostatitis (GP) with epithelioid cells, multinucleated giant cells and infiltration lymphocytes (Figure-3).

Patients with mycobacterial GP are mostly asymptomatic, with elevated PSA levels and indurated prostate at digital rectal examination, but because of its relative rarity, the MRI characteristics of infective GP caused by Mycobacterium tuberculosis or after intravesical BCG instillations have not been described extensively and only a few cases have been reported (1, 2). GP is found in approximately 75% of patients after intravesical administration of BCG for superficial bladder cancer (3). Despite the consistent ability of mpMRI to identify lesions suspicious for prostate cancer (PCa), there are other entities which can cause a false-positive result as GP, bacterial prostatitis or malacoplakia. GP chronic pattern is common, with low mean ADC value <1000, decreased signal on the ADC map images and isointense or decreased signal on high-b-value imaging (b>1200) (4) that could be differentiated by the intralesional ADC values, significantly lower in PCa, as suggested by Rais-Bahrami (5). Recent studies also demonstrated an acute pattern (less than six months prior to the mpMRI) of GP lesions, with lower signal intensity on T2-weighted imaging (T2WI) (1), decreased signal on the ADC map images (3) and increased signal on high-b-value imaging (5), that is indistinguishable from aggressive prostate cancer.
